# Psychometric evaluation of the revised Olweus Bully/Victim Questionnaire in Japanese adolescents

**DOI:** 10.3389/fpubh.2026.1812071

**Published:** 2026-06-02

**Authors:** Ekachaeryanti Zain, Atsunori Sugimoto, Hiroyuki Kasahara, Kiyohiro Yoshinaga, Masaya Ootake, Yusuke Kuwana, Faisal Budisasmita Paturungi Parawansa, Yuichiro Watanabe, Naoki Fukui, Jun Egawa, Shuken Boku

**Affiliations:** 1 Department of Psychiatry, Niigata University Graduate School of Medical and Dental Sciences, Niigata, Japan; 2 Department of Community Psychiatric Medicine, Niigata University Graduate School of Medical and Dental Sciences, Niigata, Japan; 3 Department of Psychiatry, Niigata Psychiatric Center, Nagaoka, Japan; 4 Department of Psychiatry, Niigata Prefectural Shibata Hospital, Shibata, Japan; 5 Department of Neurology, Faculty of Medicine, Hasanuddin University, Makassar, Indonesia; 6 Department of Psychiatry, Uonuma Kikan Hospital, Niigata, Japan; 7 Medical Education Center, Faculty of Medicine, Niigata University, Niigata, Japan

**Keywords:** adolescents, aggression, bullying, factor analysis, psychosocial difficulties, scale validation, victimization

## Abstract

**Background:**

School bullying is a major public health concern and is associated with adolescent mental health status. Although validated instruments exist to assess bullying victimization and witnessing in Japan, the psychometric validity of perpetration measures remains unclear. This study aimed to evaluate the psychometric validity of the revised Olweus Bully/Victim Questionnaire (OBVQ-R), focusing particularly on its perpetration subscale.

**Methods:**

Data from a cross-sectional online self-report survey were collected from 339 students in Grades 4–9 from four private schools in Niigata Prefecture, Japan. Participants completed the Japanese versions of the OBVQ-R, the Japan Ijime Scale (JaIS), and the Strengths and Difficulties Questionnaire (SDQ). We conducted reliability analysis, confirmatory factor analysis, and structural equation modeling.

**Results:**

From the original 39-item OBVQ-R, all 10 victimization items were retained, whereas 6 perpetration items were included after excluding 4 items due to insufficient variability and low variance. The 16-item OBVQ-R total scale demonstrated acceptable internal consistency overall, with satisfactory reliability for the victimization subscale but lower reliability for the perpetration subscale. Despite this, the two-factor OBVQ model showed excellent fit [comparative fit index (CFI) = 0.995, root mean square error of approximation (RMSEA) = 0.018]. The JaIS model also demonstrated excellent fit (CFI = 0.973, RMSEA = 0.038). OBVQ-R perpetration was strongly associated with JaIS perpetration (*β* = 0.70) and significantly associated with conduct problems, hyperactivity, and peer problems, as well as lower prosocial behavior.

**Conclusion:**

Although the OBVQ-R victimization subscale demonstrated satisfactory reliability, the perpetration subscale showed limited internal consistency despite clear construct relevance and meaningful associations with psychosocial difficulties. These findings highlight the importance of assessing perpetration alongside victimization and witnessing, and the need for culturally sensitive adaptation of perpetration subscales to reduce reporting reluctance in the Japanese context and thus inform bullying prevention and intervention strategies.

## 1 Introduction

Bullying in schools affects child and adolescent mental health and has become an important public health concern with a relatively high prevalence across countries (1, 2). A meta-analysis of 80 international studies suggested global prevalence rates of 36% for bullying victimization and 35% for perpetration, which may vary across contexts (3). In Japan, a large-scale, school-based survey found that the prevalence of bullying victimization, witnessing, and perpetration occurring every 2–3 months was 35.8, 32.8, and 11.8%, respectively (4). Evidence has consistently suggested strong associations between bullying involvement, including victimization and perpetration, and a wide range of mental health problems, including depression, anxiety, post-traumatic stress disorder, substance and behavioral addictions, self-harm, and suicidal ideation or attempts (5, 6). In Japan, school bullying has been identified as one of the main contributors to adolescent suicide (7).

Bullying is a complex and multifaceted phenomenon that has been defined in various ways across cultures, legal frameworks, and academic disciplines. In scientific literature, school bullying is generally defined as unwanted and repeated aggressive behavior characterized by a real or perceived power imbalance, in which more powerful children oppress less powerful peers over time (8–10). Such behaviors may involve physical, verbal, emotional, or psychological harm. In contrast, the Act on the Promotion of Measures to Prevent Bullying in Japan defines school bullying (*ijime*) as “the conduct of influencing a student mentally or physically, including through the internet, by another student(s) who has a certain personal relationship with the student, such that the victim feels mental anguish or physical pain” (11). Unlike the scientific definition, the Japanese legal definition does not require intent, repetition, or power imbalance, but instead emphasizes the victim’s subjective experience of harm.

The Olweus Bully/Victim Questionnaire—revised version (OBVQ-R) (12, 13) is one of the most widely used instruments for assessing bullying involvement and includes multi-item subscales for victimization and perpetration. The OBVQ and the latter OBVQ-R have demonstrated good psychometric properties in several populations (14–19). However, previous research in Japan has focused more extensively on victimization, whereas the validity of multi-item perpetration assessment remains insufficiently examined. The Japan Ijime Scale (JaIS) (4) was developed and validated to assess bullying experiences in 4th–9th grade students in Japan, with particular emphasis on victimization and witnessing. The JaIS assesses the same nine categories of bullying as the OBVQ-R, including physical bullying, verbal bullying, social exclusion or isolation, having money or things taken or damaged, lies and false rumors, being forced to do things, racial bullying, sexual bullying, and cyberbullying (4, 12). However, unlike the OBVQ-R, the JaIS includes only a single item to assess perpetration because of ethical constraints imposed by the local Board of Education at the time of the study (4). Direct assessment of perpetration was considered sensitive in the Japanese context due to concerns regarding stigmatization, social desirability bias, and cultural norms emphasizing social harmony and avoidance of self-disclosure. While this single-item perpetration scale approach may reduce participant discomfort, it may also limit comprehensive evaluation of perpetration behaviors compared with multi-item assessments. Therefore, comparative evaluation of the OBVQ-R and JaIS is essential to provide a valuable opportunity to examine the feasibility and validity of multi-item perpetration assessment in Japanese adolescents.

Although the adverse mental health consequences of bullying victimization are well established, evidence regarding bullying perpetration remains comparatively limited (20, 21). Emerging research suggests that bullying perpetration is more likely to occur among youth with neurodevelopmental or psychiatric conditions, particularly those exhibiting internalizing and externalizing symptoms (22). Longitudinal evidence further indicates that mental health difficulties may precede involvement in bullying, including becoming a perpetrator (23). Collectively, these findings suggest that involvement in bullying, whether as a victim, perpetrator, or witness, may be bidirectionally associated with mental health problems, serving both as a cause and a consequence (24). Bullying involvement has therefore been conceptualized as a multidimensional construct including victimization, perpetration, and witnessing experiences, which may overlap while remaining distinguishable domains (25, 26). Based on this framework, instruments assessing bullying experiences are expected to demonstrate convergent relationships across similar domains while preserving construct validity. This highlights the importance of assessing perpetration alongside victimization and witnessing to better understand bullying-related psychopathology.

In the present study, we aimed to evaluate the psychometric validity of the OBVQ-R in Japanese adolescents, with particular focus on the perpetration subscale. Specifically, we examined (1) the theory-driven construct validity and internal consistency of the OBVQ-R, (2) its concurrent validity in relation to the JaIS, and (3) its external validity by examining associations with psychosocial difficulties, as measured by the Strengths and Difficulties Questionnaire—parent report (SDQ-p). Based on prior literature and the cultural context of bullying in Japan, we hypothesized that the victimization subscale would demonstrate higher internal consistency and stronger construct validity than the perpetration subscale. In contrast, we expected the perpetration subscale to show lower reliability, while maintaining construct relevance through meaningful associations with related psychosocial measures, reflecting its context-dependent validity. The aim of clarifying the appropriateness of multi-item perpetration measurement in Japanese school populations was to inform future research and support the development of effective bullying prevention and intervention strategies.

## 2 Materials and methods

### 2.1 Study design and participants

This study was part of Phase 1 of the *International Collaborative Research in Ijime (Bullying) Prevention and Intervention* (ICoRIPI), a multinational research project conducted across six countries (Indonesia, Japan, Libya, Nigeria, Poland, and Taiwan). Phase 1 focuses on the use of standardized instruments that will be used in subsequent phases, including multinational large-scale surveys and randomized controlled trials of bullying prevention and intervention programs.

The present study used a cross-sectional, online, self-report design and was conducted between June and July 2025. All procedures involving human participants were conducted in accordance with the principles of the Declaration of Helsinki (World Medical Association, 2024 revision). Ethical approval for the Phase 1 study was obtained from the ethics committee of Niigata University (Approval Number: 2023–0320).

Participants were students in Grades 4–9 (corresponding to ages 9–15 years) and their parents or legal guardians residing in the same household. Students were recruited from two private elementary schools and two private junior high schools in Niigata Prefecture, Japan. Participants were recruited from private schools due to institutional accessibility and feasibility of data collection, as these schools were able to provide timely approval and support for research implementation. Written permission to conduct the study was obtained from all participating schools. Participation was voluntary. Informed consent was obtained electronically from parents or guardians, and assent was obtained from the students. All participants were fully informed about the study objectives, procedures, and their rights as research participants.

All data were anonymized, and participant privacy was rigorously protected throughout and after the study, given the sensitive nature of bullying-related information. Electronic data were stored on password-protected computers and via encrypted Dropbox storage, with access restricted to authorized members of the research team.

Students were excluded if they had difficulty reading or writing in Japanese or had moderate to severe intellectual disabilities that could impair comprehension of the questionnaires, based on information provided by teachers or parents/guardians. We also excluded students with known severe mental disorders, including but not limited to psychotic disorders, severe mood disorders, severe autism spectrum disorders, severe attention-deficit/hyperactivity disorder with behavioral disturbances, severe anxiety or obsessive-compulsive disorder, severe eating disorders, or those in an acute suicidal state.

**Figure 1:**
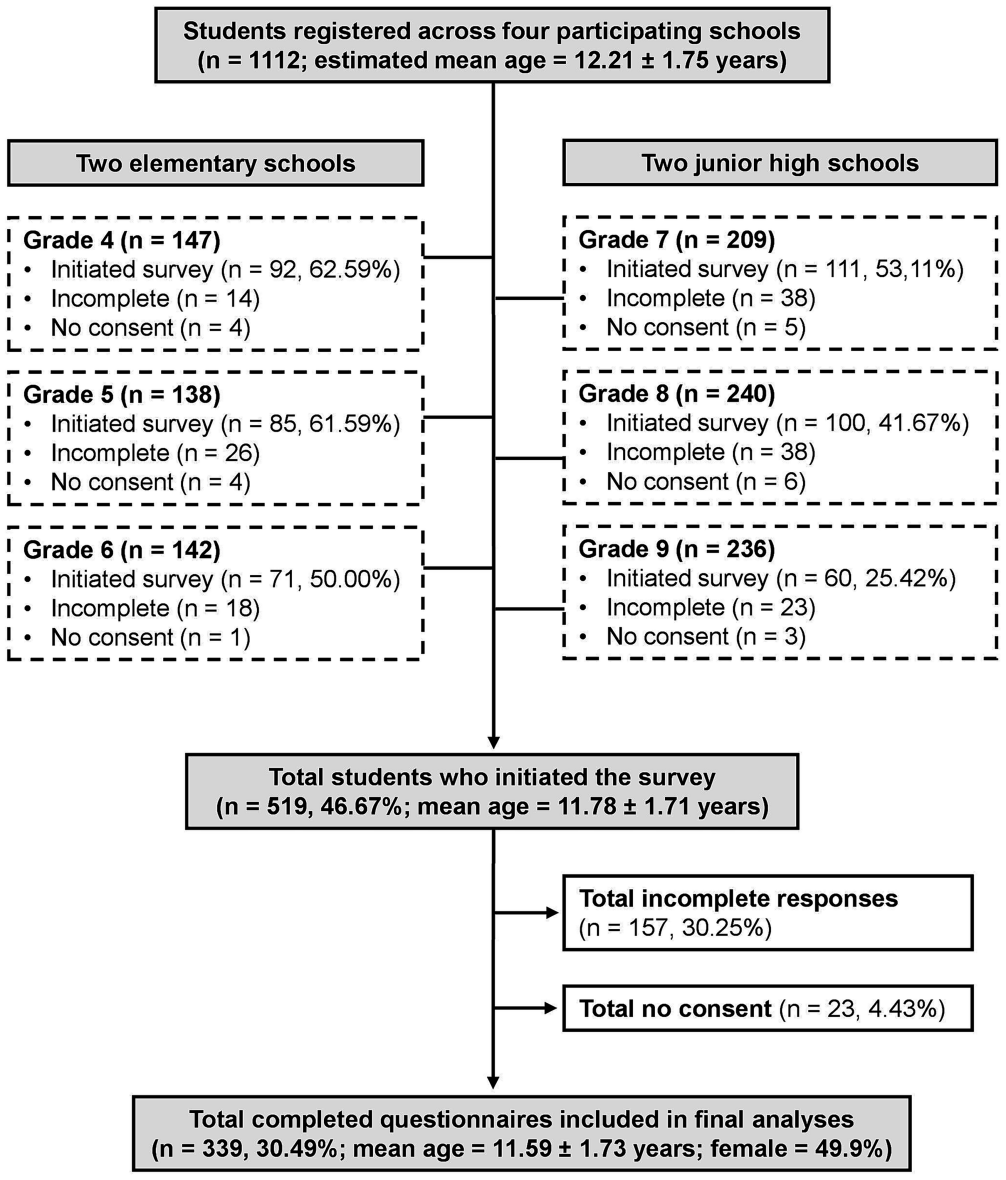
Flowchart of participant recruitment and inclusion.

Of the 1,112 students registered across participating schools, 519 (46.67%) responded to the survey. Of those respondents, 23 (4.43%) subsequently withdrew by not providing consent and 157 (30.25%) did not complete the survey. The remaining 339 (30.49%) students completed all questionnaires without missing data and were included in the final analyses (Figure 1). Age information was collected from the student who initiated the survey; however, individual age data were unavailable for all registered students who did not initiate the survey. Therefore, the overall mean age was estimated using grade-weighted estimators based on stratified population summaries (27). The estimated standard deviation was derived using a pooled variance formula that accounts for both within-grade variability and between-grade differences (28). Gender information was collected via an OBVQ-R item and was thus available only for students who completed the OBVQ-R. As a result, potential gender imbalance at earlier stages of recruitment could not be assessed.

### 2.2 Measures

All measures used in this study were self-report questionnaires administered via an online, web-based platform; no clinician diagnostic confirmation was obtained. Student participants completed the OBVQ-R and the JaIS, while parents or guardians completed the SDQ-p.

The item order of the 39-item OBVQ-R followed that of the original instrument, and the item orders of the 19-item JaIS and 25-item SDQ-p followed those of the previously validated Japanese versions. For student participants, the questionnaires were compiled into a single online survey and presented in a fixed sequence, with the JaIS administered first, followed by the OBVQ-R. It was therefore assumed that most students completed the questionnaires in the order provided.

#### 2.2.1 Olweus Bully/Victim Questionnaire—revised version (OBVQ-R)

The OBVQ-R (13) is a self-report instrument designed to assess involvement in bullying within the previous 2–3 months through two domains: victimization (being bullied) and perpetration (bullying others). The questionnaire consists of 39 items rated on a 5-point Likert scale ranging from 0 to 4 (0 = “It hasn’t happened to me in the last two months,” 1 = “It happened to me only once or twice in the last two months,” 2 = “It happened to me 2–3 times a month,” 3 = “It happened to me about once a week,” and 4 = “It happened to me several times a week”).

The OBVQ-R has demonstrated good psychometric properties in Norwegian school populations, with reported internal consistencies ranging from 0.80 to 0.90 (12). In Japan, the OBVQ-R has been translated and used in research (29); however, it has not been formally validated or standardized. In the present study, the OBVQ-R was translated from the original English version into Japanese following the World Health Organization guidelines for the translation and cultural adaptation of instruments (30, 31). This process included forward-translation by two bilingual experts in the child and adolescent psychiatric field, back-translation, pre-testing, and revision based on linguistic and cultural feedback. Permission to translate and standardize the instrument was obtained from the original author (12).

Previous research has shown that the OBVQ-R comprises two subscales that assess victimization (items 5, 6, 7, 8, 9, 10, 11, 12, 12a, 13) and perpetration (items 25, 26, 27, 28, 29, 30, 31, 32, 32a, 33). The items on each subscale measure different types of bullying, including physical bullying, verbal bullying, social exclusion or isolation, theft or damage of belongings, spreading false rumors, threats or coercion, racial bullying, sexual bullying, cyberbullying, and other bullying (16).

#### 2.2.2 Japan Ijime scale (JaIS)

The JaIS (4) is a 19-item self-report questionnaire developed in Japanese to assess bullying experiences among students. It comprises three components: a victimization subscale, a witnessing subscale, and a single item assessing perpetration. The victimization and witnessing subscales each consist of nine items that measure different types of bullying. The types of bullying assessed are consistent with those of the OBVQ-R, except that the JaIS does not include an “other bullying” category.

The JaIS assesses experiences occurring within the previous 2–3 months. Responses for the victimization subscale and the perpetration item are recorded on a 5-point Likert scale ranging from 0 (“Nothing in the past 2–3 months”) to 4 (“Several times a week in the past 2–3 months”). The witnessing subscale uses binary response options: 0 (“I have never seen or heard anything”) and 1 (“I have seen or heard something”). The JaIS has demonstrated satisfactory validity for the assessment of victimization and witnessing in Japanese school settings; however, because of ethical constraints at the time of its development, the single-item perpetration measure has not been validated (4). In the present study, the factorial structure of the JaIS was examined using structural equation modeling (SEM), and the scale was used to assess the concurrent validity of the OBVQ-R.

#### 2.2.3 Strengths and difficulties questionnaire—parent report (SDQ-p)

The SDQ (32) is a 25-item screening instrument designed to assess social, emotional, and behavioral functioning in children and adolescents aged 4–17 years. In this study, we used the Japanese parent-report version (SDQ-p) to ensure that the survey was suitable for all participants’ ages, which ranged from 9 to 15 years. The SDQ consists of five subscales: Emotional Symptoms, Conduct Problems, Hyperactivity/Inattention, Peer Problems, and Prosocial Behavior; each subscale contains five items.

Responses are rated on a 3-point Likert scale (0 = “not true,” 1 = “somewhat true,” 2 = “certainly true”). Total possible subscale scores range from 0 to 10. The Japanese version of the SDQ has demonstrated a five-factor structure consistent with the original English version (33), as well as good reliability in Japanese children and adolescents (Cronbach’s *α* = 0.77) (34).

### 2.3 Data analysis

Demographic characteristics of the participants were summarized using means and standard deviations. Bullying prevalence and characteristics measured by the JaIS and OBVQ-R, as well as psychosocial difficulties assessed by the SDQ-p, were presented descriptively in tables using counts, percentages, means, and standard deviations. The SDQ-p scores were evaluated using the Kruskal–Wallis test due to non-normal distributions and comparisons across multiple independent groups with unequal sample sizes. Overall significance was set at *p* < 0.006 based on Bonferroni correction for eight tests. Subsequently, post-hoc pairwise comparisons were conducted using Dunn’s test, which is appropriate following a Kruskal–Wallis test, with Holm correction (significant at *p* < 0.05) applied to control for Type I error while maintaining statistical power to assess group differences related to bullying experiences.

The internal consistency reliability of the OBVQ-R and JaIS was assessed using Cronbach’s alpha (*α*) and McDonald’s omega (*ω*); the latter was selected as it is a more robust reliability estimate for potentially multidimensional constructs (35). The Cronbach’s alpha (α) values were described as satisfactory (0.58–0.97) or not satisfactory (0.4–0.55) with its 95% confidence interval (CI) (36). The Kaiser–Meyer–Olkin (KMO) measure of sampling adequacy and Bartlett’s test of sphericity were conducted to assess the suitability of the data for factor analysis. A KMO value greater than 0.60 was considered acceptable (37), and a significant Bartlett’s test (*p* < 0.05) indicated that the data were appropriate for factor analysis (38). Confirmatory factor analysis (CFA) was conducted to separately examine the construct validity of the OBVQ-R and JaIS. CFA models were estimated using the weighted least squares mean and variance-adjusted (WLSMV) estimator, implemented using diagonally weighted least squares (DWLS) (39). Mardia’s test indicated violation of multivariate normality (*p* < 0.05) (40). Therefore, SEM analyses were conducted using the robust weighted least squares estimator (WLSMV/DWLS), which is appropriate for ordinal and non-normally distributed data. Model fit was evaluated using standard indices, including the comparative fit index (CFI ≥ 0.95), Tucker–Lewis index (TLI ≥ 0.95), and root mean square error of approximation (RMSEA ≤ 0.06) (41). Measurement invariance across gender and grade was explored using multi-group confirmatory factor analysis.

SEM was subsequently performed to examine the concurrent validity of the OBVQ-R in relation to the JaIS (42) and the external validity of bullying-related constructs in relation to SDQ subscales, using the same above-mentioned model fit criteria. Standardized path coefficients (*β*) were reported, with statistical significance set at *p* < 0.05.

All statistical analyses were conducted using IBM SPSS Statistics for Mac OS Version 29.0.0 IBM Corp. (43) and R version 4.3.3. R Core Team (44). CFA and SEM analyses were performed using the lavaan package in R (45).

## 3 Results

### 3.1 Descriptive statistics

Data from 339 students and their parents who completed all questionnaires and had no missing values were included in the analyses. Demographic characteristics of student participants by grade and sex are presented in Table 1. The parent participants were between 34 and 68 years old (mean age ± standard deviation = 46.00 ± 5.02).

**Table 1:** Demographic characteristics of student participants based on grade, age, and sex (*N* = 339).

Grade	Girls	Boys	Total
4th (aged 9–10 years)	33 (9.7%)	41 (12.1%)	74 (21.8%)
5th (aged 10–11 years)	27 (8.0%)	28 (8.3%)	55 (16.2%)
6th (aged 11–12 years)	27 (8.0%)	25 (7.4%)	52 (15.3%)
7th (aged 12–13 years)	40 (11.8%)	28 (8.3%)	68 (20.1%)
8th (aged 13–14 years)	27 (8.0%)	29 (8.6%)	56 (16.5%)
9th (aged 14–15 years)	15 (4.4%)	19 (5.6%)	34 (10.0%)
All grades	169 (49.9%)	170 (50.1%)	339 (100.0%)

Data are numbers of students (*n*) and percentages (%).

Based on scores on the previously validated JaIS, the prevalence of any bullying-related experiences during the last 2–3 months across all grades was as follows: 34.2% of students reported victimization (29.8% victims only and 4.4% bully/victims), 32.8% reported witnessing at least one type of bullying, and 5.6% reported perpetration (1.18% perpetrators only and 4.42% bully/victims) (Table 2).

**Table 2:** Prevalence of students with any bullying-related experiences (based on Japan Ijime scale scores) in the last 3 months by grade (*N* = 339).

Grade	Victimization		Perpetration			Witnessing	
	Never	Ever	Never	Ever (bully/victim)	Ever (bully only)	Never	Ever
4th (aged 9–10 years)	47 (63.5%)	27 (36.5%)	72 (97.3%)	2 (2.7%)	0 (0.0%)	46 (62.2%)	28 (37.8%)
5th (aged 10–11 years)	35 (63.6%)	20 (36.4%)	55 (100.0%)	0 (0.0%)	0 (0.0%)	42 (76.4%)	13 (23.6%)
6th (aged 11–12 years)	29 (55.8%)	23 (44.2%)	44 (84.6%)	5 (9.6%)	3 (5.8%)	25 (48.1%)	27 (51.9%)
7th (aged 12–13 years)	47 (69.1%)	21 (30.9%)	64 (94.1%)	4 (5.9%)	0 (0.0%)	53 (77.9%)	15 (22.1%)
8th (aged 13–14 years)	42 (75.0%)	14 (25.0%)	52 (92.9%)	3 (5.4%)	1 (1.8%)	47 (83.9%)	9 (16.1%)
9th (aged 14–15 years)	23 (67.6%)	11 (32.4%)	33 (97.1%)	1 (2.9%)	0 (0.0%)	28 (82.4%)	6 (17.6%)
All grades	223 (65.8%)	116 (34.2%)	320 (94.4%)	15 (4.4%)	4 (1.2%)	241 (71.1%)	98 (28.9%)

Data are numbers of students (*n*) and percentages (%).

Table 3 presents the prevalence of bullying by type based on JaIS scores. The most prevalent forms of victimization were verbal bullying (15.6%), spreading lies or false rumors (12.1%), and physical bullying (11.2%) (Table 3). The most frequently reported forms of witnessing were verbal bullying (13.0%) and physical bullying (10.6%).

**Table 3:** Prevalence of bullying victimization and witnessing by type and frequency (based on Japan Ijime Scale scores) in the last 3 months among grade 4–9 students (*N* = 339).

Bullying types and frequency	Physical bullying	Verbal bullying	Social exclusion or isolation	Having money or things taken or damaged	Lies and false rumors	Being forced to do things	Racial bullying	Sexual bullying	Cyberbullying
Victimization (item)	(1)	(2)	(3)	(4)	(5)	(6)	(7)	(8)	(9)
Never	301 (88.8%)	286 (84.4%)	313 (92.3%)	317 (93.5%)	298 (87.9%)	318 (93.8%)	321 (94.7%)	324 (95.6%)	332 (97.9%)
Ever	38 (11.2%)	53 (15.6%)	26 (3.7%)	22 (6.5%)	41 (12.1%)	21 (6.2%)	18 (5.3%)	15 (2.1%)	7 (2.1%)
Only once or twice	27 (8.0%)	37 (10.9%)	17 (5.0%)	20 (5.9%)	33 (9.7%)	15 (4.4%)	13 (3.8%)	11 (3.2%)	7 (2.1%)
Two or three times a month	6 (1.8%)	11 (3.2%)	3 (0.9%)	1 (0.3%)	3 (0.9%)	3 (0.9%)	2 (0.6%)	3 (0.9%)	0 (0.0%)
Once a week	3 (0.9%)	2 (0.6%)	1 (0.3%)	0 (0.0%)	3 (0.9%)	2 (0.6%)	2 (0.6%)	1 (0.3%)	0 (0.0%)
Several times a week	2 (0.6%)	3 (0.9%)	5 (1.5%)	1 (0.3%)	2 (0.6%)	1 (0.3%)	1 (0.3%)	0 (0.0%)	0 (0.0%)
Witnessing (item)	(11)	(12)	(13)	(14)	(15)	(16)	(17)	(18)	(19)
Have never seen bullying	303 (89.4%)	295 (87.0%)	312 (92.0%)	309 (91.2%)	319 (94.1%)	333 (98.2%)	320 (94.4%)	332 (97.9%)	329 (97.1%)
Have seen bullying	36 (10.6%)	44 (13.0%)	27 (8.0%)	30 (8.8%)	20 (5.9%)	6 (1.8%)	19 (5.6%)	7 (2.1%)	10 (2.9%)

Data are frequency of occurrence (*n*) and percentages (%).

Table 4 summarizes bullying prevalence by type based on OBVQ-R scores. Verbal bullying was the most prevalent form for both victimization (12.7%) and perpetration (5.9%).

**Table 4:** Prevalence of bullying victimization and perpetration by type and frequency (based on Olweus Bully/Victim Questionnaire–revised version scores) in the last 3 months among grade 4–9 students (*N* = 339).

Bullying types and frequency	Verbal bullying	Social exclusion or isolation	Physical bullying	Lies and false rumors	Having money or things taken or damaged	Being forced to do things	Racial bullying	Sexual bullying	Cyberbullying	Other bullying
Victimization (item)	(5)	(6)	(7)	(8)	(9)	(10)	(11)	(12)	(12a)	(13)
Never	296 (87.3%)	322 (95.0%)	317 (93.5%)	325 (95.9%)	325 (95.9%)	324 (95.6%)	332 (97.9%)	332 (97.9%)	332 (97.9%)	322 (95.0%)
Ever	43 (12.7%)	17 (5.0%)	22 (6.5%)	14 (4.1%)	14 (4.1%)	15 (4.4%)	7 (2.1%)	7 (2.1%)	7 (2.1%)	17 (5.0%)
Only once or twice	32 (9.4%)	13 (3.8%)	16 (4.7%)	12 (3.5%)	14 (4.1%)	14 (4.1%)	6 (1.8%)	5 (1.5%)	7 (2.1%)	15 (4.4%)
Two or three times a month	6 (1.8%)	1 (0.3%)	3 (0.9%)	1 (0.3%)	0 (0.0%)	0 (0.0%)	1 (0.3%)	1 (0.3%)	0 (0.0%)	1 (0.3%)
Once a week	1 (0.3%)	0 (0.0%)	1 (0.3%)	0 (0.0%)	0 (0.0%)	0 (0.0%)	0 (0.0%)	1 (0.3%)	0 (0.0%)	1 (0.3%)
Several times a week	4 (1.2%)	3 (0.9%)	2 (0.6%)	1 (0.3%)	0 (0.0%)	1 (0.3%)	0 (0.0%)	0 (0.0%)	0 (0.0%)	0 (0.0%)
Perpetration (item)	(25)	(26)	(27)	(28)	(29)	(30)	(31)	(32)	(32a)	(33)
Never	319 (94.1%)	330 (97.3%)	333 (98.2%)	337 (99.4%)	334 (98.5%)	337 (99.4%)	339 (100%)	338 (99.7%)	339 (100%)	338 (99.7%)
Ever	20 (5.9%)	9 (2.7%)	6 (1.8%)	2 (0.6%)	5 (1.5%)	2 (0.6%)	0 (0.0%)	1 (0.3%)	0 (0.0%)	1 (0.3%)
Only once or twice	18 (5.3%)	6 (1.8%)	5 (1.5%)	0 (0.0%)	5 (1.5%)	0 (0.0%)	0 (0.0%)	1 (0.3%)	0 (0.0%)	1 (0.3%)
Two or three times a month	1 (0.3%)	1 (0.3%)	0 (0.0%)	0 (0.0%)	0 (0.0%)	0 (0.0%)	0 (0.0%)	0 (0.0%)	0 (0.0%)	0 (0.0%)
Once a week	0 (0.0%)	0 (0.0%)	0 (0.0%)	0 (0.0%)	0 (0.0%)	0 (0.0%)	0 (0.0%)	0 (0.0%)	0 (0.0%)	0 (0.0%)
Several times a week	1 (0.3%)	2 (0.6%)	1 (0.3%)	0 (0.0%)	0 (0.0%)	0 (0.0%)	0 (0.0%)	0 (0.0%)	0 (0.0%)	0 (0.0%)

Data are frequency of occurrence (*n*) and percentages (%).

Table 5 shows the SDQ-p scores categorized by bullying experiences based on OBVQ-R scores. Mean SDQ-p scores differed across bullying experience groups. The Kruskal–Wallis test demonstrated significant group differences in peer problems, internalizing problems, and total difficulties (*p* = 0.002, 0.002, and 0.003, respectively). Post-hoc Dunn’s tests with Holm correction indicated that for peer problems, students in both the victim only (*p* = 0.033) and bully/victim (*p* = 0.017) groups showed significantly higher scores than students who had never experienced bullying. Internalizing problems were significantly higher for students in the victim only group compared with those who had never experienced bullying (*p* = 0.018). Additionally, total difficulties scores were significantly higher among bully/victim students than among those who had never experienced bullying (*p* = 0.021).

**Table 5:** Strengths and difficulties questionnaire scores reported by parents based on OBVQ-R categorization of any bullying-related experiences (*N* = 339).

Variables	Group 1: never experienced any bullying (*n* = 243)	Group 2: ever (victim only) (*n* = 65)	Group 3: ever (bully only) (*n* = 15)	Group 4: ever (bully/victim) (*n* = 16)	*p* overall	Post-hoc 1 *vs* 2	1 *vs* 3	1 *vs* 4	2 *vs* 3	2 *vs* 4	3 *vs* 4
Emotional problems	1.16 ± 1.771	1.78 ± 2.183	0.40 ± 0.737	1.56 ± 1.931	0.019	—	—	—	—	—	—
Conduct problems	1.35 ± 1.369	1.71 ± 1.400	1.47 ± 1.356	2.00 ± 1.461	0.076	—	—	—	—	—	—
Hyperactivity/inattention	2.21 ± 1.944	2.45 ± 2.187	2.33 ± 2.637	3.38 ± 2.156	0.159	—	—	—	—	—	—
Peer problems	1.42 ± 1.383	2.05 ± 1.709	1.33 ± 1.447	2.38 ± 1.147	**0.002**	**0.033**	0.765	**0.017**	0.327	0.327	0.072
Prosocial behavior	6.85 ± 2.048	7.15 ± 2.116	6.07 ± 2.404	5.69 ± 2.272	0.045	—	—	—	—	—	—
Internalizing	2.58 ± 2.639	3.83 ± 3.277	1.73 ± 1.534	3.94 ± 2.670	**0.002**	**0.018**	0.640	0.081	0.081	0.640	0.081
Externalizing	3.57 ± 2.850	4.15 ± 3.083	3.80 ± 3.342	5.38 ± 3.222	0.103	—	—	—	—	—	—
Total difficulties score	6.15 ± 4.363	7.98 ± 5.378	5.53 ± 4.224	9.31 ± 4.191	**0.003**	0.066	0.600	**0.021**	0.293	0.293	0.065

OBVQ-R, Olweus Bully/Victim Questionnaire—revised version.

Data are mean ± standard deviation.

Bold values indicate statistically significant results. Overall significance was set at *p* < 0.006 based on Bonferroni correction for eight tests. Group differences were examined using the Kruskal–Wallis test owing to non-normal distributions and unequal group sizes. Post-hoc pairwise comparisons were conducted using Dunn’s test with Holm correction (significant at *p* < 0.05).

### 3.2 Internal consistency reliability, KMO test, and Bartlett’s test of sphericity

To evaluate the internal consistency reliability of the OBVQ-R, four perpetration items were excluded because of insufficient variability. Items 31 and 32a were removed because of zero variance, which did not allow the reliability test, and items 32 and 33 were excluded owing to extremely low variance (Table 3). The reduced 16-item set was retained because it demonstrated better psychometric performance compared to the 18-item version, which showed lower reliability [Cronbach’s *α* = 0.706, 95% CI (0.658–0.749); McDonald’s *ω* cannot be estimated]. The remaining 16 items demonstrated satisfactory internal consistency [Cronbach’s *α* = 0.712, 95% CI (0.665–0.755); McDonald’s *ω* = 0.715, 95% CI (0.665–0.755)]. Subscale analyses indicated satisfactory internal consistency for the victimization subscale (Cronbach’s *α* = 0.708, 95% CI [0.660–0.753]; McDonald’s *ω* = 0.725, 95% CI [0.660–0.753]). However, the internal consistency of the perpetration subscale was not satisfactory [Cronbach’s α = 0.461, 95% CI (0.367–0.545); McDonald’s ω = 0.444, 95% CI (0.367–0.545)].

The JaIS demonstrated satisfactory internal consistency for the total scale [Cronbach’s α = 0.769, 95% CI (0.731–0.803); McDonald’s ω = 0.770, 95% CI (0.731–0.803)]. The reliability was satisfactory for both the victimization subscale [Cronbach’s α = 0.701, 95% CI (0.650–0.746); McDonald’s ω = 0.712, 95% CI (0.650–0.746)] and the witnessing subscale [Cronbach’s α = 0.649, 95% CI (0.591–0.703); McDonald’s ω = 0.652, 95% CI (0.591–0.703)].

In the present study, the KMO value was 0.66, and Bartlett’s test was significant [*χ^2^*(120) = 1213.24, *p* < 0.001].

### 3.3 Confirmatory factor analysis (CFA)

**Figure 2:**
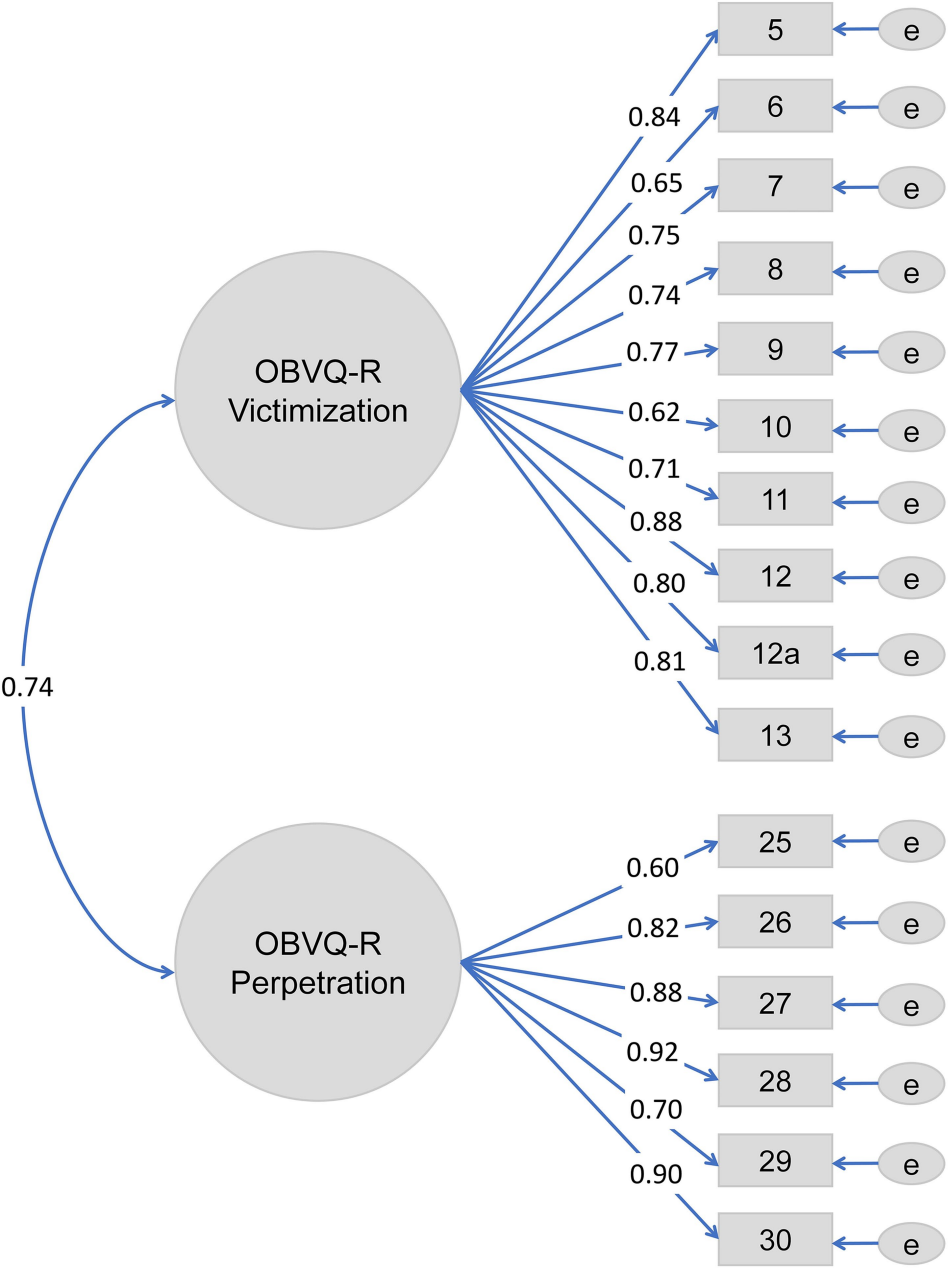
Confirmatory factor analysis of the OBVQ-R. The model depicts a two-factor structure with victimization and perpetration specified as correlated latent variables based on 16 retained items. Bold lines show significant paths (all *p* < 0.0001), with the estimate (*β*) representing the standardized path coefficient. OBVQ-R, Olweus Bully/Victim Questionnaire—revised version.

Drawing on theory-driven models and previously reported factor structures from studies conducted in Chile and Bangladesh (16, 18), CFA was performed to examine a two-factor model of the OBVQ-R comprising correlated latent variables of victimization and perpetration. The results confirmed that the 16-item OBVQ-R two-factor correlated model demonstrated excellent fit to the data, with RMSEA (90% CI) = 0.018 (0.001–0.034), CFI = 0.995, and TLI = 0.995 (Figure 2). Victimization was significantly and positively correlated with perpetration (*β* = 0.74, *p* < 0.0001). The CFA results provide evidence of construct validity for the OBVQ-R, as the hypothesized two-factor structure demonstrated excellent model fit, supporting the theoretical distinction between victimization and perpetration and indicating that the observed items adequately represent the underlying constructs.

Gender invariance analyses of OBVQ were conducted only for the victimization subscale using dichotomized responses because stable estimation of the perpetration subscale across gender groups was not possible due to extremely low endorsement frequencies and sparse categorical distributions. Configural invariance demonstrated excellent fit (CFI = 1.000, TLI = 1.005, RMSEA = 0.000), while metric invariance was generally supported (CFI = 0.989, TLI = 0.987, RMSEA = 0.042). Grade-level invariance could not be reliably estimated because several items demonstrated no variance within some grade subgroups.

**Figure 3:**
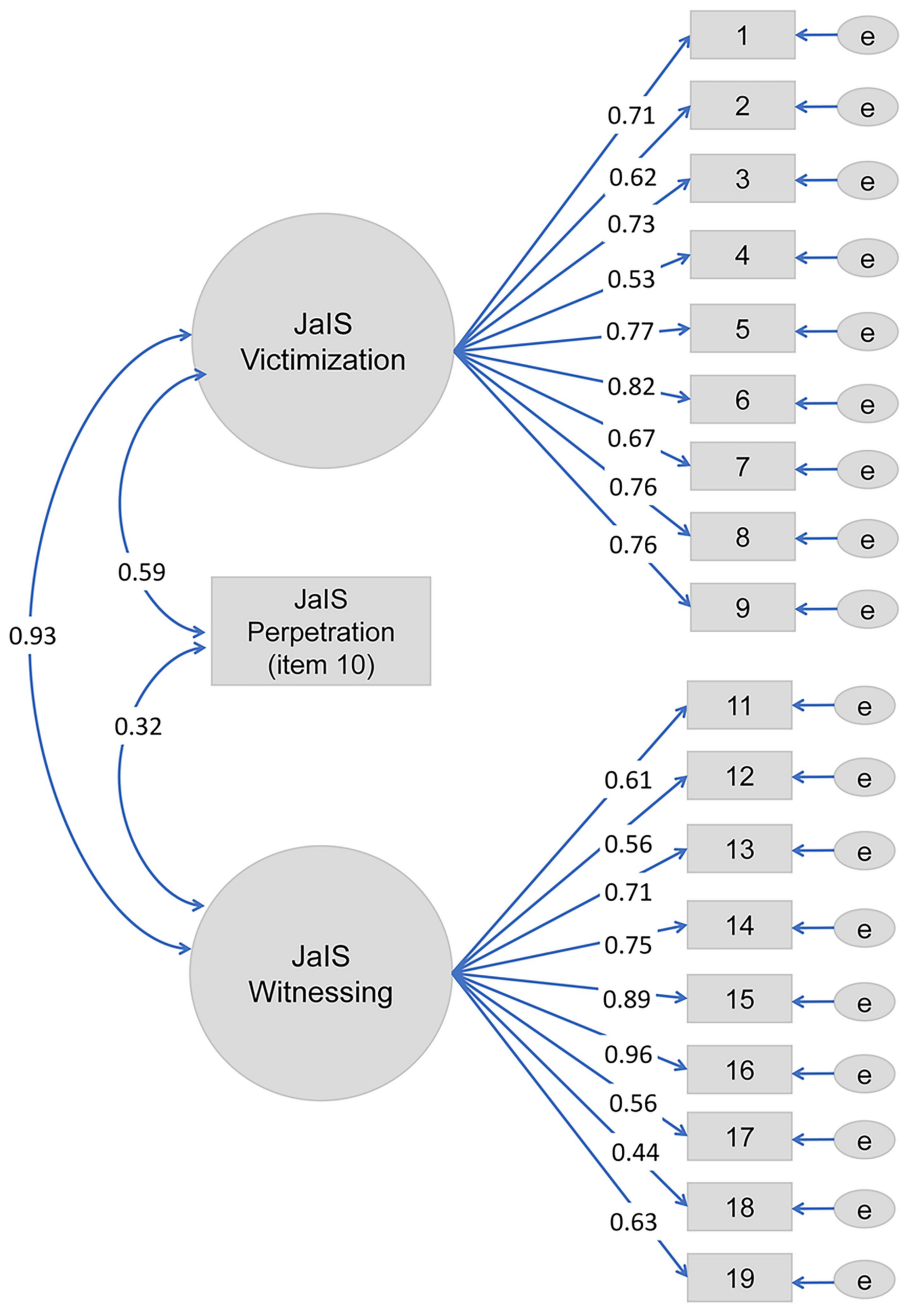
Confirmatory factor analysis of the JaIS. The model depicts victimization and witnessing as latent variables and perpetration as a single observed variable. Bold lines indicate significant paths (all *p* < 0.0001), with estimates (β) representing standardized path coefficients. JaIS, Japan Ijime Scale.

For the JaIS, CFA was conducted based on the previously validated 19-item structure, with victimization and witnessing modeled as latent variables and perpetration included as a single observed item. The model demonstrated excellent fit, with RMSEA (90% CI) = 0.038 (0.028–0.048), CFI = 0.973, and TLI = 0.969 (Figure 3). Victimization was significantly positively correlated with witnessing (*β* = 0.93, *p* < 0.0001) and perpetration (*β* = 0.59, *p* < 0.0001). Witnessing was also significantly positively correlated with perpetration (*β* = 0.32, *p* < 0.0001).

### 3.4 Structural equation modeling (SEM)

**Figure 4:**
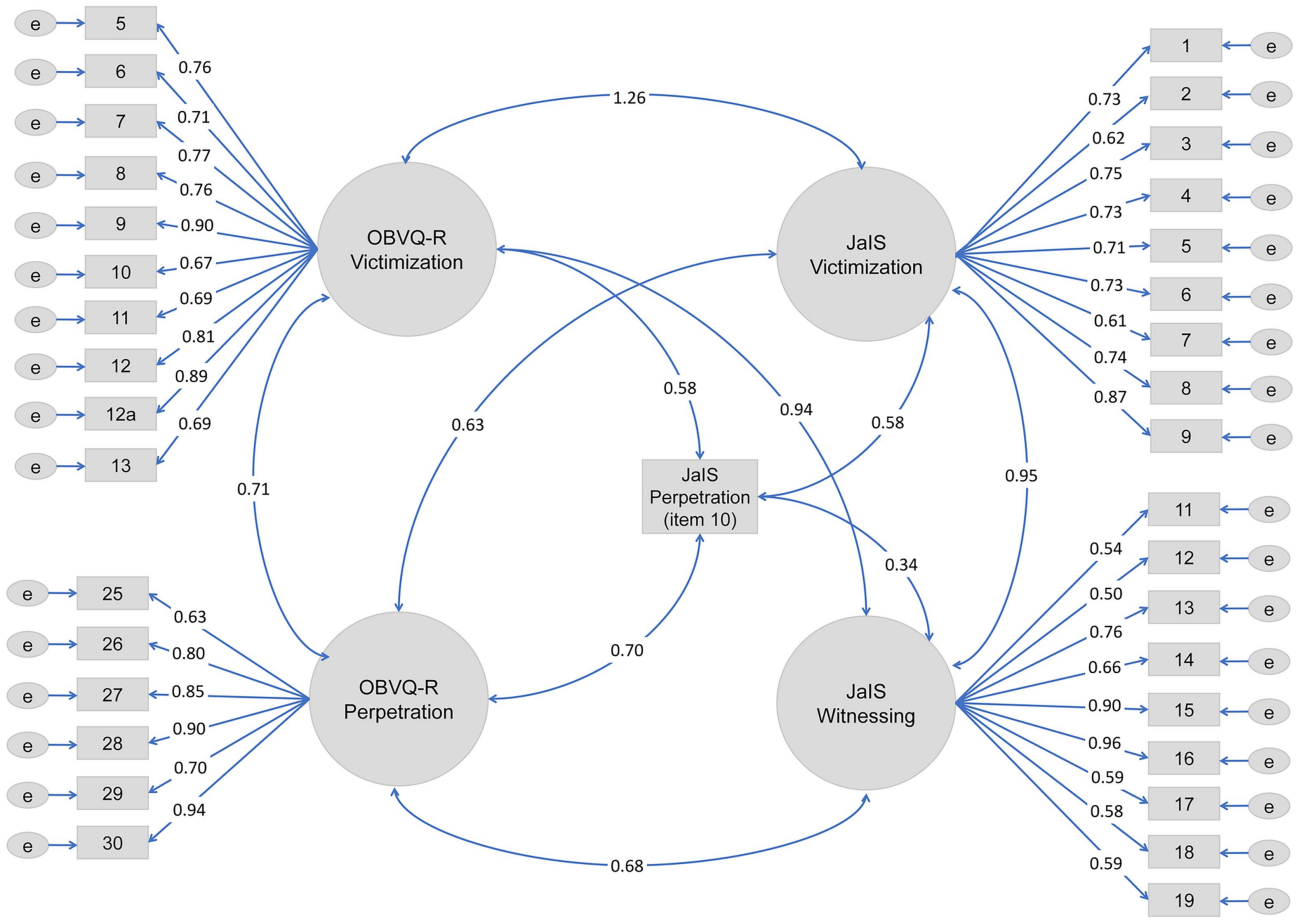
Structural equation model examining concurrent validity between the OBVQ-R and JaIS. The model depicts associations between victimization and perpetration constructs of the OBVQ-R and corresponding constructs of the JaIS. Bold lines indicate significant paths (all *p* < 0.001), with estimates (β) representing standardized path coefficients. JaIS, Japan Ijime Scale; OBVQ-R, Olweus Bully/Victim Questionnaire—revised version.

SEM was conducted to examine the concurrent validity between the OBVQ-R and the previously validated JaIS in the Japanese population. The proposed path model demonstrated excellent fit to the data, with RMSEA (90% CI) = 0.049 (0.044–0.053), CFI = 0.975, and TLI = 0.973 (Figure 4). This SEM model was over-identified. The model included 35 observed variables, yielding 630 unique variance–covariance elements [35 variances + 35(35−1)/2 covariance’s]. The number of free parameters to be estimated was 44, resulting in 586 degrees of freedom. The model indicated a strong linear association between OBVQ-R victimization and JaIS victimization (*β* = 1.26, *p* < 0.001), reflecting the conceptual similarity of these subscales. OBVQ-R perpetration was also significantly associated with JaIS perpetration (*β* = 0.70, *p* < 0.001).

**Figure 5:**
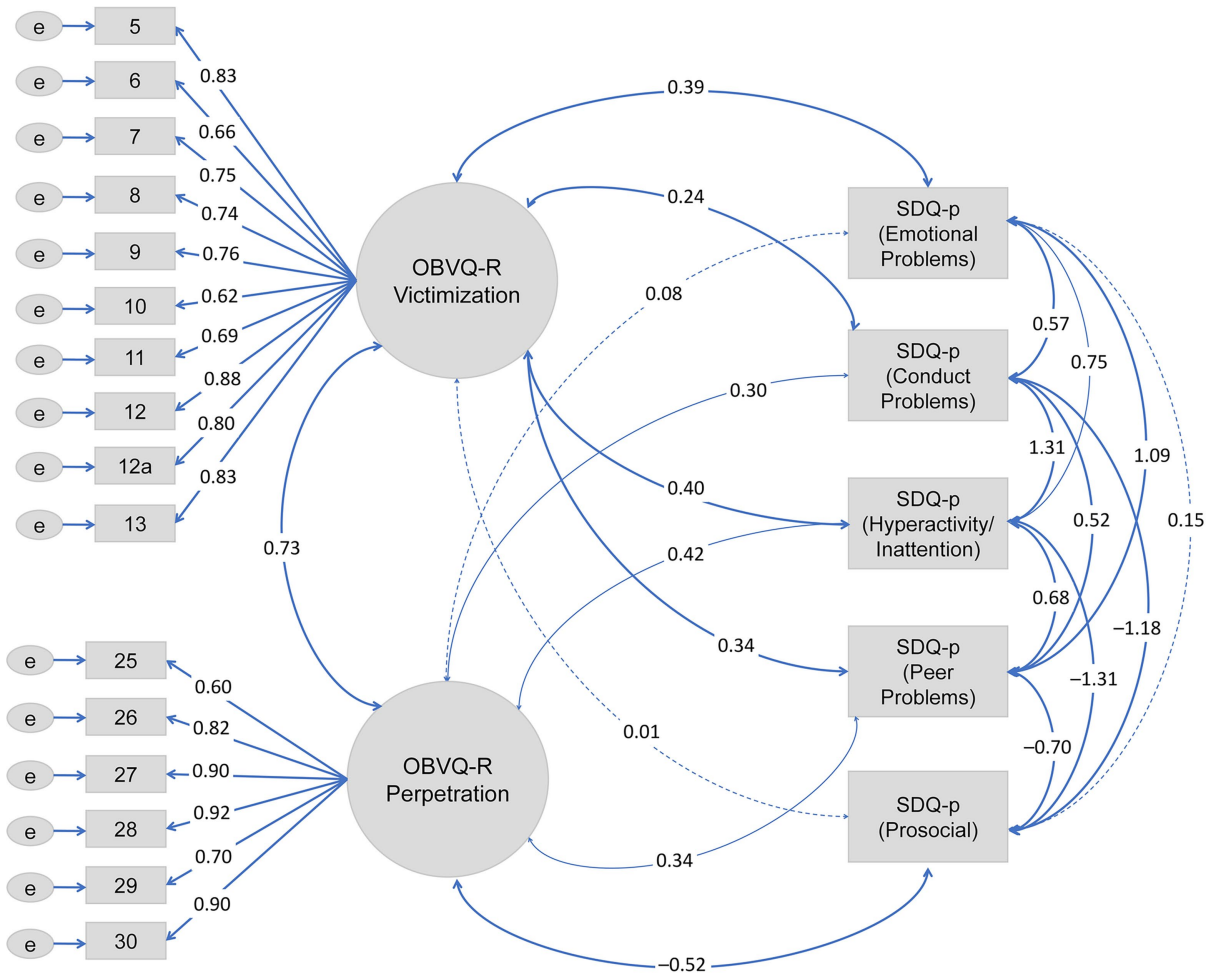
Structural equation model examining the external validity of the OBVQ-R with the SDQ-p. The model depicts associations between OBVQ-R victimization and perpetration latent variables and observed subscales of the SDQ-p. Bold lines indicate significant paths (*p* < 0.001), fine lines indicate significant paths (*p* < 0.05), and dashed lines indicate non-significant paths (*p* = ns). Estimates (*β*) represent standardized path coefficients. OBVQ-R, Olweus Bully/Victim Questionnaire—revised version; SDQ-p, Strengths and Difficulties Questionnaire—parent report.

To further examine the external validity of the OBVQ-R, SEM was conducted with OBVQ-R victimization and perpetration modeled as latent variables and the five subscales of the SDQ-p included as observed variables. This model demonstrated excellent fit to the data, with RMSEA (90% CI) = 0.018 (0.001–0.031), CFI = 0.974, and TLI = 0.968 (Figure 5). The SEM shown in Figure 5 was also over-identified. The model included 21 observed variables, yielding 231 unique variance–covariance elements [21 variances + 21(21−1)/2 covariance’s]. The number of free parameters to be estimated was 37, resulting in 194 degrees of freedom. From this model, victimization was significantly and positively associated with emotional symptoms, conduct problems, hyperactivity/inattention, and peer problems (*β* = 0.39, 0.24, 0.40, and 0.34, respectively; all *p* < 0.001). Perpetration was significantly positively associated with conduct problems, hyperactivity/inattention, and peer problems (*β* = 0.30, 0.42, and 0.34, respectively; all *p* < 0.05) and significantly negatively associated with prosocial behavior (*β* = −0.52, *p* < 0.001).

## 4 Discussion

The present study evaluated the psychometric validity of the OBVQ-R in Japanese adolescents, with particular focus on the perpetration subscale, using the JaIS and the SDQ-p for concurrent and external validation.

The descriptive findings indicated that the prevalence of bullying victimization and witnessing as measured by the JaIS in the present study (Table 2) was comparable to that reported in a large-scale, school-based survey of 2,334 students in Japan conducted by Osuka et al. (4). This may indicate that the overall pattern of bullying experiences in the present sample is consistent with previous large-scale data, and may confirm the representativeness of the sample for evaluating the validity of the OBVQ-R in the Japanese context.

This study is the first to report the psychometric properties of the OBVQ-R among adolescents in Japan. Consistent with previous studies conducted in Norway, Chile, Bangladesh, and Germany, the present findings support the unidimensionality of the victimization and perpetration subscales and confirm a two-factor correlated latent structure for the OBVQ-R (Figure 2) (12, 16–18). Although the internal consistency of the 16-item Japanese OBVQ-R and its victimization subscale was satisfactory (*α* and *ω* > 0.70), the perpetration subscale demonstrated unsatisfactory internal consistency in the present study. Furthermore, among the perpetration items analyzed, four items related to racial bullying, sexual bullying, cyberbullying, and other types of bullying (items 31, 32, 32a, and 33) were excluded owing to insufficient variability (Table 4), a pattern that has also been reported in a previous study (18). Despite the limited internal consistency, the perpetration subscale retained meaningful associations with JaIS perpetration and psychosocial difficulties, supporting its construct relevance. These findings suggest that the reduced subscale captures important aspects of bullying perpetration, although its measurement may remain constrained by contextual and reporting factors in the Japanese setting.

The present study also demonstrated, for the first time, a well-fitting model of the JaIS comprising two latent factors (victimization and witnessing) and a single observed perpetration item, confirming its construct validity (Figure 3). The internal consistency of the JaIS total scale and its victimization and witnessing subscales was satisfactory (*α* and *ω* > 0.70), consistent with previous findings obtained using item-response theory (4). However, the internal consistency of perpetration could not be evaluated as it was a single-item measure.

The strong associations observed in the path model between OBVQ-R victimization and JaIS victimization further support convergent validity (Figure 4), as both scales assess closely related constructs, differing mainly in the inclusion of an “other bullying” category in the OBVQ-R. The OBVQ-R perpetration subscale was also strongly associated with the single-item perpetration measure of the JaIS. Although the JaIS assesses perpetration using only one item, its association with the OBVQ-R perpetration subscale (comprising six retained perpetration items) supports the convergent validity of the OBVQ-R perpetration indicators.

Although the reliability of the JaIS single-item perpetration measure could not be evaluated and the internal consistency of the OBVQ-R perpetration subscale was also limited, the structural model indicated that OBVQ-R perpetration showed stronger associations with victimization and witnessing in the JaIS than did the single-item perpetration measure. This suggests that multi-item assessment of perpetration may provide a more comprehensive evaluation of bullying involvement, particularly considering the interrelations between victimization, perpetration, and witnessing experiences.

Although the perpetration subscale of the OBVQ-R has demonstrated satisfactory reliability in studies from Chile, Bangladesh, and Germany (*α*/*ω* > 0.80) (16–18), its reliability was low in the present Japanese sample (*α* = 0.419, *ω* = 0.443). Among the 5.6% of students who reported perpetration in the present study, most (4.42%) were classified as bully–victims, suggesting that self-reports may be more strongly oriented toward victimization experiences than toward perpetration alone. This tendency may be affected by the Japanese legal definition of *ijime*, which emphasizes the victim’s subjective experience of harm (11). In addition, students may cognitively justify their own aggressive behaviors by framing them as retaliation in response to perceived harm (46), thereby reducing the likelihood of identifying themselves as perpetrators. Furthermore, in peer contexts where social exclusion or peer rejection is salient, some students may minimize or rationalize perpetration by aligning themselves with perpetrators as a form of self-protection to avoid becoming victims (47). Taking all these factors into consideration in this context, the low internal consistency of self-reported perpetration is more likely to reflect ethical, cultural, and institutional constraints on measurement rather than inadequacy of the underlying construct, thereby limiting the validity of multi-item perpetration as assessed by the OBVQ-R. Consistent with this interpretation, the assessment of perpetration in Japanese schools is ethically sensitive; notably, during the development of the JaIS, perpetration was restricted to a single item by the local Board of Education owing to ethical concerns (4), which may suppress endorsement and restrict response variability.

In addition, the Japanese legal and cultural conceptualization of *ijime* emphasizes the plurality of perpetrators within a collectivistic social context, which often results in diffused responsibility and ambiguity regarding a single “main perpetrator” (48, 49). Under such circumstances, students may be reluctant to label themselves as perpetrators, as doing so may be perceived as self-stigmatizing and socially alienating (49). Furthermore, individuals tend to underestimate or reinterpret their own aggressive behaviors and may resist identification with the “perpetrator” role, contributing to social desirability bias in self-report measures (50). Taken together, these findings suggest that while bullying perpetration is a meaningful construct, its expression and self-report may be highly context-dependent, and shaped by cultural norms emphasizing social harmony, avoidance of self-disclosure, and sensitivity to social evaluation.

Despite its limited reliability, the OBVQ-R perpetration subscale demonstrated theoretically consistent associations with several psychopathology domains as measured by the SDQ-p (Figure 5). In the present study, perpetration was positively associated with conduct problems, consistent with previous findings (4, 51). Although the developmental trajectories of bullying behavior and conduct problems are distinct, these behaviors often co-occur across childhood, likely owing to shared risk factors (51, 52).

Perpetration was also positively associated with hyperactivity/inattention and peer problems. Previous research has reported associations between bullying involvement and attention-deficit/hyperactivity disorder (53), and perpetrators have been shown to experience greater peer rejection, reflected in elevated peer problems (54). In addition, our results demonstrated a negative association between perpetration and prosocial behavior. This finding is consistent with evidence highlighting the antisocial nature of bullying behaviors (52), and previous research has suggested that promoting prosocial behavior may be an effective component of bullying prevention programs (55).

Importantly, these associations should be interpreted as bidirectional rather than causal. Longitudinal studies indicate that mental health difficulties may both precede and result from involvement in bullying, including perpetration (56, 57). Taken together, our findings support the view that bullying perpetration, even within the Japanese context, constitutes an integral component of bullying-related psychopathology and should be assessed alongside victimization and witnessing.

From a measurement perspective, the present findings suggest that direct use of the self-report, multi-item perpetration subscales of the OBVQ-R may be insufficient for Japanese school populations. Culturally sensitive refinement of perpetration items, such as emphasizing participation in indirect or group bullying, adding contexts of retaliation and self-defense, and adjusting wording to reduce perceptions of self-stigmatization, may improve the reliability of perpetration assessment in this context. Alternatively, a single-item indicator, such as that used in the JaIS (4), may offer a pragmatic approach for large-scale screening when ethical or contextual constraints limit disclosure. Accordingly, further research using a group-oriented and collective cultural perspective on perpetration is warranted, and could inform future school-based prevention and intervention programs in Japan.

This study has several strengths, including the use of theory-driven analyses and a rigorous process for translating the OBVQ-R into Japanese. Nevertheless, several limitations should be acknowledged. The sample size was relatively small, and the response rate was relatively low compared to the number of eligible participants. In addition, the sample was limited to four private schools in a single prefecture, which may limit generalizability. Students in private schools may differ from the broader adolescent population in Japan in terms of socioeconomic background, academic environment, and school climate, which may influence both the prevalence and expression of bullying. Participants with severe mental disorders were excluded to ensure reliable self-reporting and reduce participant burden. However, this may have led to the underrepresentation of adolescents with more severe clinical conditions who may be at higher risk of bullying involvement. Future studies should consider more inclusive approaches, such as the use of adapted assessment tools or additional ethical safeguards, to enable participation of these populations while ensuring their well-being. Exploratory factor analysis and item-response theory were not conducted. Because the OBVQ-R is a well-established, theory-based instrument, we prioritized a confirmatory, theory-driven approach rather than exploratory analyses. In addition, the sample size, although sufficient for CFA, was relatively small and not optimal for stable item-response theory parameter estimation, particularly for low-frequency perpetration incidents. Therefore, caution is warranted when generalizing these findings to the wider population. Furthermore, reliance on self-report measures introduces the possibility of social desirability bias, especially for sensitive and potentially stigmatizing behaviors such as bullying perpetration within Japan’s harmony-oriented collective culture. Future studies using larger and more diverse samples, culturally adapted perpetration items, and complementary qualitative approaches, such as in-depth interviews, may help to further elucidate item-level performance and cultural sensitivity in the detailed assessment of bullying perpetration in the Japanese context.

## 5 Conclusion

In conclusion, the OBVQ-R demonstrated acceptable validity for assessing bullying victimization among Japanese adolescents, whereas the perpetration subscale showed limited reliability despite demonstrating construct relevance and meaningful associations with psychosocial difficulties. These findings underscore the importance of assessing perpetration alongside victimization and witnessing, and highlight the need for culturally sensitive adaptation of perpetration subscales that may reduce reluctance in the Japanese context. Further research using newly developed and culturally appropriate perpetration items in larger and more diverse Japanese samples is warranted to improve assessment reliability and support effective bullying prevention and intervention strategies.

## Data Availability

The datasets presented in this article are not readily available because the underlying individual-level data cannot be shared publicly, as participant consent for data sharing was not obtained. Requests to access the datasets should be directed to ekazain@med.niigata-u.ac.jp.
